# PrP^C^-facilitated cell signaling activates phospholipase Cɣ1 and triggers an Arc/Arg3.1 response in mouse and iPSC-derived human neurons

**DOI:** 10.1016/j.stemcr.2026.102924

**Published:** 2026-05-14

**Authors:** Daniel Ojeda-Juarez, Gail Funk, Daniel B. McClatchy, Emily Richards, Alexander J. Rajic, Katrin Soldau, Michael D. Geschwind, Xu Chen, John R. Yates, Steven L. Gonias, Christina J. Sigurdson

**Affiliations:** 1 Department of Pathology, University of California, San Diego, La Jolla, CA 92093, USA; 2 Department of Integrative Structural and Computational Biology, The Scripps Research Institute, La Jolla, CA 92037, USA; 3 Department of Neurology, Weill Institute for Neurosciences, Memory and Aging Center, University of California, San Francisco, San Francisco, CA 94158, USA; 4 Department of Neurosciences, University of California, San Diego, La Jolla, CA 92093, USA; 5 Department of Pathology, Immunology, and Microbiology, University of California, Davis, Davis, CA 95616, USA

**Keywords:** protein misfolding, Alzheimer’s disease, proteomics, neurodegeneration, prion disease, amyloid, Ca^2+^ signaling, EGFR, PLC-γ1, Creutzfeldt-Jakob disease

## Abstract

Synapse loss is an early feature of prion disease, yet the underlying drivers are poorly understood. We recently found evidence of neuronal hyperactivity and synaptic loss in prion-infected mice. Herein, we identified increased Arc/Arg3.1 in patients with prion disease, suggesting heightened neuronal activity also occurs in the human prion-affected brain. To determine the signaling events initiated by prion aggregates (PrP^Sc^), we developed a disease model in which human iPSC-derived excitatory neurons are stimulated with a PrP^Sc^-mimetic antibody, POM1, that binds cellular prion protein (PrP^C^). Within 2 h of POM1 exposure, we detected an Arc/Arg3.1 response together with transcriptomic changes previously reported in prion-infected mice. We identified altered phosphorylation of PLC-γ1, ERK1/2, and EGFR as additional PrP^C^-triggered cell signaling events. These results suggest that PrP^C^ ligands, including PrP^Sc^, trigger rapid signaling events linked to neuronal hyperactivity in human neurons, and indicate PLC-γ1 as a potential therapeutic target.

## Highlights

• Triggering PrP^C^ induces an Arc/Arg3.1 response in human iPSC-derived neurons
• Triggering PrP^C^ increases phosphorylated PLC-γ1 and ERK1/2
• Blocking dynamin-mediated endocytosis prevents Arc induction and ERK1/2 phosphorylation
• Phosphorylated PLC-γ1 is also increased in the brain of prion-affected mice

## Teaser

Ojeda-Juárez and colleagues report that triggering PrP^C^ in human iPSC-derived neurons (iNs) with a PrP^Sc^-mimetic increases Arc/Arg3.1, phosphorylated ERK1/2, and phosphorylated PLC-γ1 within 2 h. They observe similar alterations in prion-infected brain. These findings suggest that pathogenic ligands of PrP^C^ can rapidly increase neuronal activity and support the utility of iNs in revealing PrP^C^-linked signaling pathways.

## Introduction

Synapse loss and neuronal hyperactivity occur early in Alzheimer’s disease (AD), prior to neuronal loss and hypoactivity (Targa Dias Anastacio et al., 2022; Terry et al., 1991). Evidence of neuronal activity in AD includes an increase in the activity-regulated cytoskeleton-associated protein, Arc/Arg3.1 (subsequently referred to as Arc), in post-mortem patient brain samples (Wu et al., 2011). Aβ oligomers also trigger an Arc response in murine neurons *in vitro* (Um et al., 2013; Wu et al., 2011). Synapse loss is also an early feature of prion-induced neurodegeneration (Belichenko et al., 2000; Brown et al., 2001; Cunningham et al., 2003; Ferrer, 2002; Gray et al., 2009; Jeffrey et al., 2000), and a persistent increase in Arc in mouse models suggests neuronal hyperactivity may similarly occur in prion disease (Ojeda-Juarez et al., 2022). The pronounced long, concave post-synapses observed in prion-infected brains further support heightened neuronal activity (Lawrence et al., 2023; Sikorska et al., 2004; Siskova et al., 2013; Tao-Cheng, 2019). Yet whether neuronal hyperactivity develops in human neurons during prion disease is challenging to assess since functional imaging studies cannot resolve synaptic alterations or signaling. Nevertheless, understanding the activity status of human neurons is key to developing therapies to preserve synapses and maintain functional neuronal circuits.

The immediate-early gene (IEG), *Arc*, is rapidly transcribed in neuronal spines by either a rise in intracellular Ca^2+^ or cyclic adenosine monophosphate (cAMP) (Lyford et al., 1995; Waltereit et al., 2001) and regulates synaptic strength to maintain network stability (Nikolaienko et al., 2018); increases in Arc protein are an early indicator of neuronal activity (Steward et al., 1998). Arc functions as a master regulator of synaptic plasticity, promoting long-term depression (LTD) by regulating synaptic clustering of α-amino-3-hydroxy-5-methyl-4-isoxazolepropionic acid receptors (AMPARs) (Shepherd et al., 2006) and promoting receptor internalization (Chowdhury et al., 2006). Arc competes with PSD95 for binding to TARPs (transmembrane AMPA receptor regulatory proteins), with TARP-bound Arc resulting in AMPAR dispersal from the postsynaptic density condensate (Chen et al., 2022), which facilitates access of endocytic machinery to AMPAR in weaker synapses (“inverse” synaptic tagging; Okuno et al., 2018) to promote homeostatic synaptic downscaling (Chowdhury et al., 2006, Zhang et al., 2015).

Prion disease is characterized by the conversion of the cellular prion protein (PrP^C^) to a pathological β-sheet-rich misfolded form, PrP^Sc^ (Kraus et al., 2021; Li et al., 2022). PrP^C^ binds prion oligomers (PrP^Sc^), as well as amyloid-β (Aβ), tau, and α-synuclein oligomers, which results in the activation of macromolecular complexes and signal induction at the post-synapse (Fang et al., 2018; Haas et al., 2016; Rosener et al., 2020; Um et al., 2012, 2013). However, as a glycosylphosphatidylinositol (GPI)-anchored protein, PrP^C^ lacks an intracellular domain and thus requires a transmembrane partner for intracellular signaling. PrP^C^ has been reported to interact with a variety of cell surface receptors, including epidermal growth factor receptor (EGFR) (Llorens et al., 2013), G protein-coupled receptors (GPCRs) (Haas et al., 2016), low-density lipoprotein receptor-related protein-1 (LRP1) (Mantuano et al., 2020), neural cell adhesion molecule 1 (NCAM1) (Schmitt-Ulms et al., 2001), and N-methyl-D-aspartate receptors (NMDARs) (Khosravani et al., 2008). In AD, Aβ oligomers (Aβo) are reported to interact with PrP^C^ and mGluR5, triggering a Ca^2+^ response that activates Fyn (Um et al., 2013), and activation of the Aβo-PrP^C^-Fyn axis leads to phosphorylation of the NMDAR subunit, GluN2B, at Y1472 (Um et al., 2012). Prion oligomers trigger PrP^C^-dependent intracellular signaling, including NMDAR-linked Ca^2+^ influx and downstream p38 MAPK activation in mouse neurons (Fang et al., 2018). Although multiple receptors may interact with PrP^C^, relatively little is known about how these interactions impact cell signaling in prion disease, particularly in the context of a human neuron.

Studies using human induced pluripotent stem cell (iPSC)-derived cells or organoids with genetic mutations in *PRNP* have demonstrated aberrant synaptic structure (Foliaki et al., 2021; Gojanovich et al., 2024), excitatory-inhibitory imbalance (Foliaki et al., 2021), and altered bioenergetics (Foliaki et al., 2023; Matamoros-Angles et al., 2018), even in the absence of proteinase K-resistant oligomers (Foliaki et al., 2020, 2021, 2023; Gojanovich et al., 2024; Matamoros-Angles et al., 2018; Smith et al., 2022). However, the acute cell signaling events that may occur within hours of prion exposure are poorly understood. To begin to understand how early PrP^C^-triggered cell signaling events drive an Arc response in human neurons, we developed a model system using human iPSC-derived excitatory neurons (iNs) stimulated with a PrP^Sc^-mimetic antibody (POM1). POM1 binds PrP^C^ (Baral et al., 2012), and POM1-treated organotypic brain slices reproduce the transcriptome of prion-infected mice (Herrmann et al., 2015) while avoiding the confound of the other molecules bound to brain-derived PrP^Sc^, including lipids (Deleault et al., 2007) and heparan sulfate (Aguilar-Calvo et al., 2020). We found that POM1-exposed iNs recapitulated the increase in Arc within 2 h. We then used proteomics, RNA sequencing (RNA-seq), and a phospho-kinase array to identify POM1-induced transcript changes and downstream cell signaling events that occurred together with the increase in Arc. Notably, there was an increase in phosphorylated phospholipase C (PLC)-γ1 (pPLC-γ1) and ERK1/2 (pERK1/2), suggestive of a Ca^2+^-mediated signaling response. Prion-infected mice also showed higher levels of pPLC-γ1, suggesting that PLC-γ1 activity is also increased *in vivo*. This rapid, powerful iPSC-based human neuron model identifies PLC-γ1 and ERK1/2 as components of an acutely activated signaling pathway triggered by a PrP^Sc^ mimetic.

## Results

### Arc is increased in the cerebral cortex of patients with genetic and sporadic prion disease

**Figure 1:**
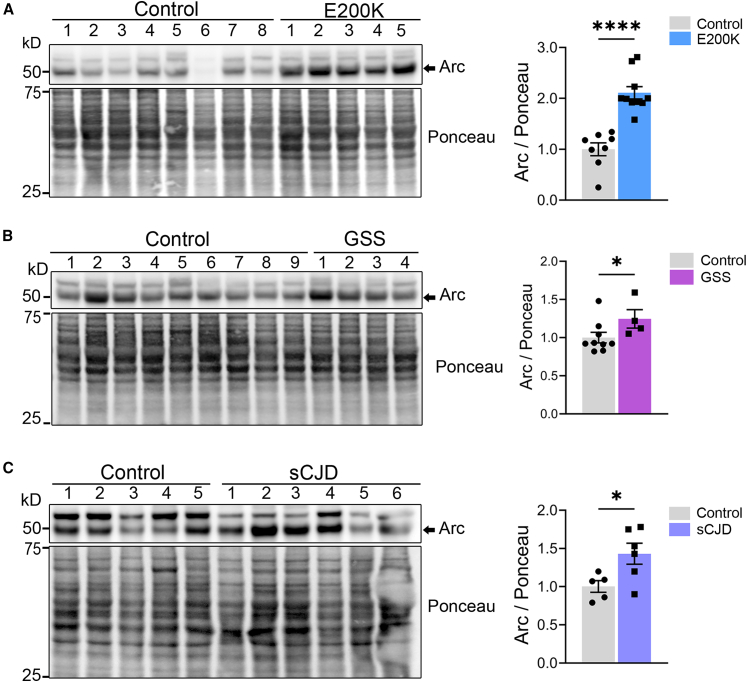
Arc is increased in the frontal cortex of human genetic and sporadic prion disease cases Immunoblots from the frontal cortex of (A) E200K familial prion disease patients (additional cases 6–10 shown in S1), (B) Gerstmann-Sträussler-Scheinker (GSS) syndrome patients, and (C) sporadic Creutzfeldt-Jakob (sCJD) patients (subtypes: MM1 and MV1 [samples 1 and 2]; MM2 [sample 3]; VV [sample 4]; MV2 [samples 5 and 6]) compared to age-matched controls (controls: *n* = 5–9; E200K: *n* = 10; GSS: P102L (*n* = 1) and F198S (*n* = 3); sCJD: *n* = 6). Data are represented as fold change compared to control samples. Shown is the mean ± SE. Mann-Whitney test. ^∗^*p* ≤ 0.05; ^∗∗∗∗^*p* ≤ 0.0001.

*Arc* is rapidly transcribed in neurons in response to increased activity (Saha et al., 2011; Steward et al., 1998). To assess Arc levels in human prion-affected brain as a proxy for neuronal activity, we quantified Arc and PK-resistant PrP^Sc^ in the frontal cortex of patients who died from genetic prion disease, either familial Creutzfeldt-Jakob disease (fCJD) (PrP-E200K; *n* = 10) or Gerstmann-Sträussler-Scheinker (GSS) (GSS: 1 PrP-P102L and 3 PrP-F198S cases; *n* = 4). Immunoblots of fCJD and GSS brain samples revealed that Arc was increased by 2.1- and 1.3-fold, respectively, compared to control brain samples (Figures 1A and 1B; Table 1; Figure S1).

**Table 1:** Number of subjects, age at the time of death, postmortem interval, and sex for control, fCJD (PrP-E200K), GSS (PrP-P102L [*n* = 1] and PrP-F198S [*n* = 3]), and sCJD (subtypes: MM1 and MV1 = 2; MM2 = 1; VV = 1; MV2 = 2) cases

**Frontal cortex E200K**			

Characteristics	Control	E200K	*p* value
Number of subjects	8	10	–
Age at death (years)	71 ± 5 (71; 63–75)	55 ± 8 (59; 43–64)	0.0002
Postmortem interval (h)	27 ± 32 (16; 2–72)	45 ± 40 (28; 0–120)	0.4
Sex (male, %)	4 (50%)	6 (60%)	–

**Frontal cortex GSS**			

Characteristics	Control	GSS	*p* value
Number of subjects	9	4	–
Age at death (years)	73 ± 5 (74; 63–79)	55 ± 21 (62; 25–72)	0.2
Postmortem interval (h)	22 ± 29 (8; 2–72)	20 ± 15 (19; 3–39)	0.8
Sex (male, %)	4 (50%)	4 (100%)	–

**Frontal cortex sCJD**			

Characteristics	Control	sCJD	*p* value
Number of subjects	5	6	–
Age at death (years)	72 ± 6 (73.5; 63–79)	68 ± 9 (69.5; 55–81)	0.3
Postmortem interval (h)	10 ± 10 (7; 2–24)	61 ± 41 (61; 8–119)	0.03
Sex (male, %)	4 (57%)	3 (50%)	–

Age is shown as mean ± standard deviation (SD) (median; range). Statistical analysis of age and postmortem interval was performed using a Welch’s *t* test.

We next measured Arc in the frontal cortex of sporadic CJD (sCJD) patients (*n* = 6). Arc was increased by 1.4-fold compared to age-matched controls (Figure 1C; Table 1), suggestive of heightened neuronal activity.

PrP^Sc^ was present in all brain samples (Figure S2), and there was no significant correlation between Arc and PrP^Sc^ levels (E200K: Spearman correlation [SC] = −0.35, *p* = 0.32; GSS: SC = 0.80, *p* = 0.33; sCJD: SC = −0.71, *p* = 0.14) (Figure S3).

### PrP^Sc^ and an anti-PrP^C^ antibody induce Arc in mouse cortical neurons within 2 h

**Figure 2:**
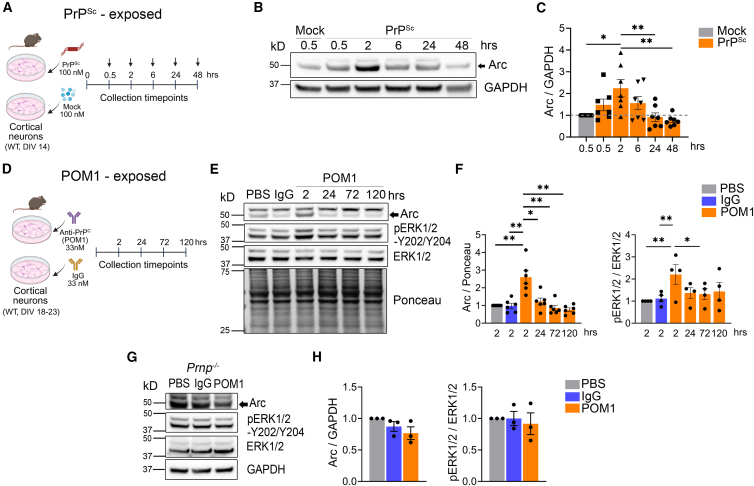
Arc is increased in mouse cortical neurons exposed to PrP^Sc^ or an anti-PrP antibody (POM1) (A) Schematic of the experiment shows PrP^Sc^ exposure of cortical neurons. (B) Immunoblots and (C) quantification of neurons (DIV 14) exposed to PrP^Sc^ or uninfected brain homogenate (mock) (*n* = 7 independent experiments). (D) Schematic of the experiment shows POM1 exposure of cortical neurons. (E) Immunoblots and (F) quantification of cortical neurons exposed to POM1 (33 nM) (DIV 18–23; *n* = 4–6 independent experiments). (G) Immunoblots and (H) quantification of POM1-treated *Prnp*^−/−^ cortical neurons (2 h) (*n* = 3 independent experiments). Data are normalized to PBS for each experiment (PBS = 1). Shown is the mean ± SE. One-way ANOVA with Tukey’s MCT. ^∗^*p* ≤ 0.05; ^∗∗^*p* ≤ 0.01.

We previously found that Arc was increased in the brain of prion-infected mice at the 40% time point (approximately 60 days post-prion exposure) (Ojeda-Juarez et al., 2022). To assess whether brain-derived PrP^Sc^ induces a rapid Arc response, we exposed cortical neurons from C57BL/6 mice (14 days *in vitro* [DIV]) to partially purified mouse PrP^Sc^ or mock brain for 0.5, 2, 6, 24, or 48 h (Figures 2A–2C). Arc levels increased by 2.2-fold at 2 h and returned to baseline by 6 h (Figures 2A–2C).

Given that partially purified PrP^Sc^ contains PrP^Sc^-bound lipids (Deleault et al., 2007) and heparan sulfate (Aguilar-Calvo et al., 2020), we next tested whether the PrP^Sc^-mimetic antibody, POM1, also generates an Arc response. POM1 binds residues 140–147 (β1-α1 loop) and K204, R208, and Q212 (α3 helix) of human PrP^C^ (Baral et al., 2012), and reproduces features of prion disease including neuronal loss, protein kinase RNA-like endoplasmic reticulum kinase (PERK) activation, production of reactive oxygen species, and reduced PIKfyve kinase levels in cultured cerebellar slices, similar to PrP^Sc^ (Herrmann et al., 2015; Lakkaraju et al., 2021). We exposed wild-type (WT) or *Prnp*^−/−^ (ZH3) (Nuvolone et al., 2016) cortical neurons (21 DIV) to POM1 or mouse IgG as a control (33 nM as used by Wu et al., 2017) for 2, 24, 72, or 120 h. Similar to PrP^Sc^, POM1 induced a 2.7-fold increase in Arc by 2 h in WT, but not *Prnp*^−/−^, neurons (Figures 2D–2H).

### Anti-PrP^C^ antibody activates extracellular-regulated kinase (ERK1/2) in mouse cortical neurons

Neuronal activity induces ERK1/2 phosphorylation and ERK1/2-dependent cell signaling (Bluthgen et al., 2017; English and Sweatt, 1996). Phosphorylated ERK1/2 is increased in the brain of prion-infected mice (LaCasse et al., 2008). To determine whether ERK1/2 was phosphorylated in neurons post-POM1 exposure, we measured ERK1/2 phosphorylated at Thr202/Tyr204 (pERK1/2). Similar to Arc, pERK1/2 was increased by 2 h in WT, but not *Prnp*^−/−^, neurons (Figures 2D–2H). Thus, POM1 induces an increase in Arc and pERK1/2 in mouse cortical neurons in a PrP^C^-dependent manner.

### PrP^C^ increases over time and localizes to the soma and synapses of human iNs

Mouse cortical neurons are a powerful tool, yet may not capture the responses of human neurons due to species-specific differences. To define the early PrP^C^-linked signaling events specific to human neurons, we differentiated human iPSCs containing a Tet-ON 3G-controlled *Ngn2* transgene (Wang et al., 2017) into excitatory neurons (iNs) (wild-type genetic background; WTC11 line; male; originally generated by Kreitzer et al., 2013). We first characterized synaptic protein and PrP^C^ expression during neuronal maturation by immunoblotting and measuring synaptophysin (presynaptic), PSD95 (postsynaptic), GluN2A and GluN2B (NMDA receptor subunits), and PrP^C^ at 0.5, 2, 4, and 6 weeks post-differentiation (Figures S4A and S4B). GluN2B, PSD95, and synaptophysin were increased at 2 and 4 weeks, and reached a plateau by 6 weeks (Figures S4A and S4B), congruent with the appearance of synapses by immunofluorescent labeling (Figure S4C). Notably, PrP^C^ continually increased from 0.5 to 6 weeks post-differentiation (Figures S4A and S4B).

To further confirm the excitatory subtype of the differentiated neurons, we performed RNA sequencing (RNA-seq) at 6 weeks post-differentiation. iNs expressed transcripts of excitatory neurons (*SLC17A6*, *SLC17A7*, *DLG4*, and *TUBB3*), glutamatergic receptors (*GLUN1* and *GRM5*), and PrP^C^ (*PRNP*) (Figure S4D), and had low to no expression of transcripts associated with pluripotent stem cells (*NANOG*, *FOXD3*, and *POU5F1*), glial cells (*GFAP*, *AIF1*, *OLIG1*, *OLIG2*, *SOX10*, *MAG*, and *MOG*), or inhibitory neurons (*GAD1* and *GAD2*) (Figure S4D).

We further characterized the morphology and localized key neuronal and synaptic proteins by immunofluorescence staining. The iNs expressed proteins of mature neurons (MAP2, Tuj1, and NeuN), vesicular glutamate transporters (vGlut), and post-synaptic structural proteins (PSD95) (Figure S4C). PrP^C^ was expressed in the cell body and in distinct puncta around the Tuj1 positive neurites, congruent with synaptic localization of PrP^C^ (Figure S4C). These findings indicate that the iNs are glutamatergic neurons expressing mature synaptic markers and PrP^C^.

### PrP^C^ stimulation of iNs with POM1 generates an Arc and pERK1/2 response by 2 h

**Figure 3:**
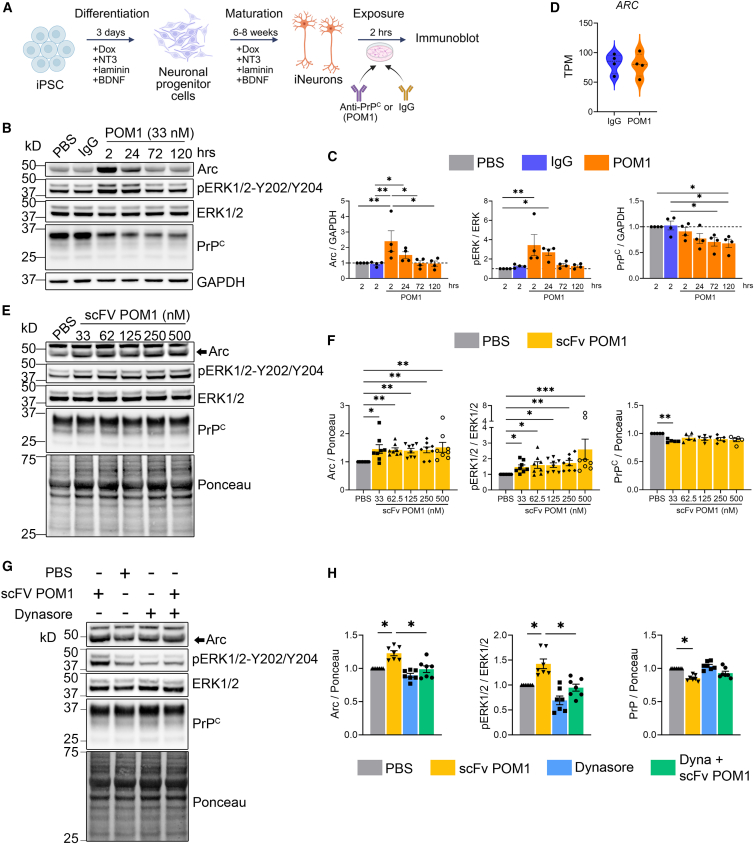
Human iNs exposed to POM1 show an early increase in Arc and phosphorylated ERK1/2 (A) Schematic timeline of differentiation, maturation, and exposure to POM1. (B) Immunoblot and (C) quantification of iNs (6–8-week old) stimulated with POM1 (33 nM) (*n* = 4 independent experiments). (D) *ARC* transcript levels in POM1-stimulated iNs as determined from RNA-seq data. *N* = 4 independent experiments. (E) Immunoblot and (F) quantification of iNs (6–8-week old) stimulated with scFvPOM1 (33–500 nM) (*n* = 6–8 independent experiments). (G) Immunoblot and (H) quantification of iNs treated for 15 min with Dynasore (100 μM) prior to svFV POM1 (33 nM) stimulation for 2 h (*n* = 7 independent experiments). For (C), (F), and (H), data are normalized to PBS for each experiment (PBS = 1). Shown is the mean ± SE. Kruskal-Wallis test with Dunn’s MCT. ^∗^*p* ≤ 0.05; ^∗∗^*p* ≤ 0.01; ^∗∗∗^*p* ≤ 0.001. (D) Mann-Whitney test.

To determine whether triggering PrP^C^ induces Arc in human neurons, we exposed iNs to POM1 or mouse IgG (33 nM) for 2 to 120 h. Both Arc and pERK1/2 levels increased by 2.4- and 3.4-fold, respectively, by 2 h, before returning to baseline levels by 72 h (Figures 3B and 3C). PrP^C^ levels, however, were significantly reduced by 72 h (Figures 3B and 3C), consistent with reports that anti-PrP antibodies induce PrP^C^ endocytosis and degradation (Perrier et al., 2004). Notably, *ARC* transcripts were unchanged by RNA-seq at 2 h, indicating that the rise in Arc protein was not due to increased transcription, but from enhanced translation or reduced degradation (Figure 3D).

To test whether IgG-mediated cross-linking of PrP^c^ was required for Arc induction and ERK1/2 phosphorylation, we exposed iNs to increasing concentrations of a single-chain variable fragment of POM1 (scFvPOM1) (33–500 nM) (Frontzek et al., 2016). Following a 2 h incubation, Arc and pERK1/2 levels increased by 45% ± 16% (mean ± SE) and 47% ± 13%, respectively, compared to control, while PrP^C^ was reduced at the lowest concentration (33 nM) (Figures 3E and 3F). These results suggest that the induction of Arc and phosphorylation of ERK1/2 do not require antibody cross-linking.

### Dynamin-dependent endocytosis is required for the Arc elevation in response to POM1

GPI-anchored PrP^C^ localizes to the cell surface (Stahl et al., 1987) and is internalized by dynamin and clathrin-mediated endocytosis (CME) or clathrin-independent endocytosis (Campana et al., 2005). Since surface PrP^C^ has not been investigated in iNs, we characterized the distribution of PrP^C^ in our iN model. We quantified surface PrP^C^ by cleaving the GPI-anchor with phosphatidylinositol-specific phospholipase C (PI-PLC). PI-PLC markedly reduced PrP^C^ levels [70% ± 13% (mean ± SE)], indicating that most PrP^C^ in iNs resides at the cell surface (Figure S5).

Next, to determine whether endocytosis of surface PrP^C^ is required for the POM1-PrP^C^-mediated signaling, we blocked dynamin-dependent endocytosis using Dynasore (100 μM) prior to the exposure of iNs to scFvPOM1 (33 nM). Inhibition of this endocytic pathway prevented Arc induction, ERK1/2 phosphorylation, and the reduction in PrP^C^ (Figures 3G and 3H). These results suggest that dynamin-mediated PrP^C^ endocytosis is required for the Arc and pERK1/2 response.

### Phospholipase C-γ1 is activated in POM1-treated iNs

**Figure 4:**
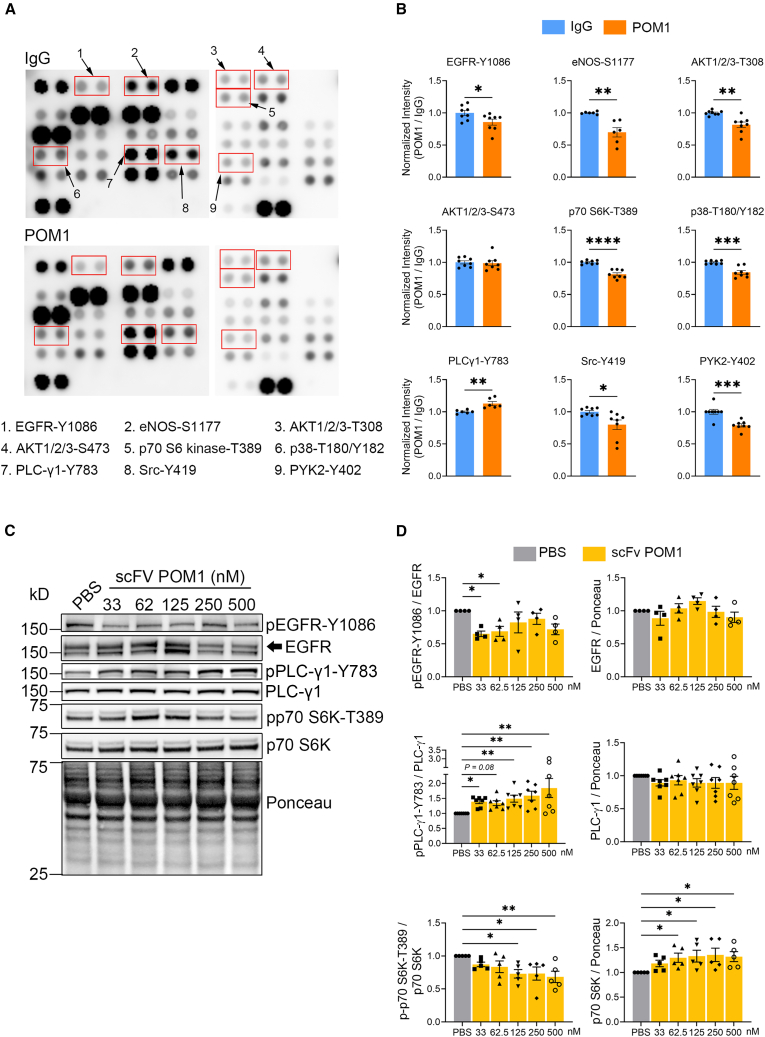
Protein phosphorylation panel reveals POM1 stimulation of PrP^C^ alters cell signaling in iNs (A) Phospho-kinase array of 37 kinases and related proteins in iNs treated with POM1 or IgG for 2 h. (B) Quantification of the array data in (A). (C) Immunoblot and (D) quantification of iNs stimulated with scFvPOM1 (33–500 nM). For (A) and (B), *n* = 4 independent experiments, each with 2 technical replicates; data are normalized to the average of the two technical replicates of IgG for each experiment. Welch’s *t* test. For (C) and (D), *n* = 4–8 independent experiments. Kruskal-Wallis test with Dunn’s MCT. Data are normalized to PBS for each experiment (PBS = 1). Shown is the mean ± SE. ^∗^*p* ≤ 0.05; ^∗∗^*p* ≤ 0.01; ^∗∗∗^*p* ≤ 0.001; ^∗∗∗∗^*p* ≤ 0.0001.

Given the POM1-triggered increase in pERK1/2, we sought to identify additional kinases that may be phosphorylated in iNs. We next measured the levels of 37 phospho-kinase phosphorylated proteins in a membrane-based antibody array of iN lysates collected 2 h after POM1 or IgG exposure. Notably, there was an increase in phosphorylated phospholipase C (pPLC)-γ1-Y783, together with a decrease in pEGFR-Y1086 (a tyrosine kinase), pAKT-T308, pp70 S6 kinase (S6K)-T389, peNOS-S1177, pp53-S15/S46/S392, pp38-T180/Y182, pSrc-Y419, and pPYK2-Y402 (Figures 4A and 4B; Figure S6).

To test whether the pPLC-γ1, pEGFR, and pp70 S6K alterations were independent of IgG-mediated cross-linking, iNs were treated with increasing concentrations of scFvPOM1 (33–500 nM) for two hours. Congruent with the phospho-kinase array, pEGFR-Y1086 and pp70 S6K-T389 were decreased (Figures 4C and 4D), with the reduction in pp70 S6K-T389 associated with an increase in total p70 S6K (Figures 4C and 4D). Interestingly, pPLC-γ1-Y783 increased in a scFvPOM1 concentration-dependent manner and was approximately 40% (33 nM) to 85% (500 nM) higher than the control (Figures 4C and 4D).

### Rapid transcriptional changes induced by triggering PrP^C^ in iNs

**Figure 5:**
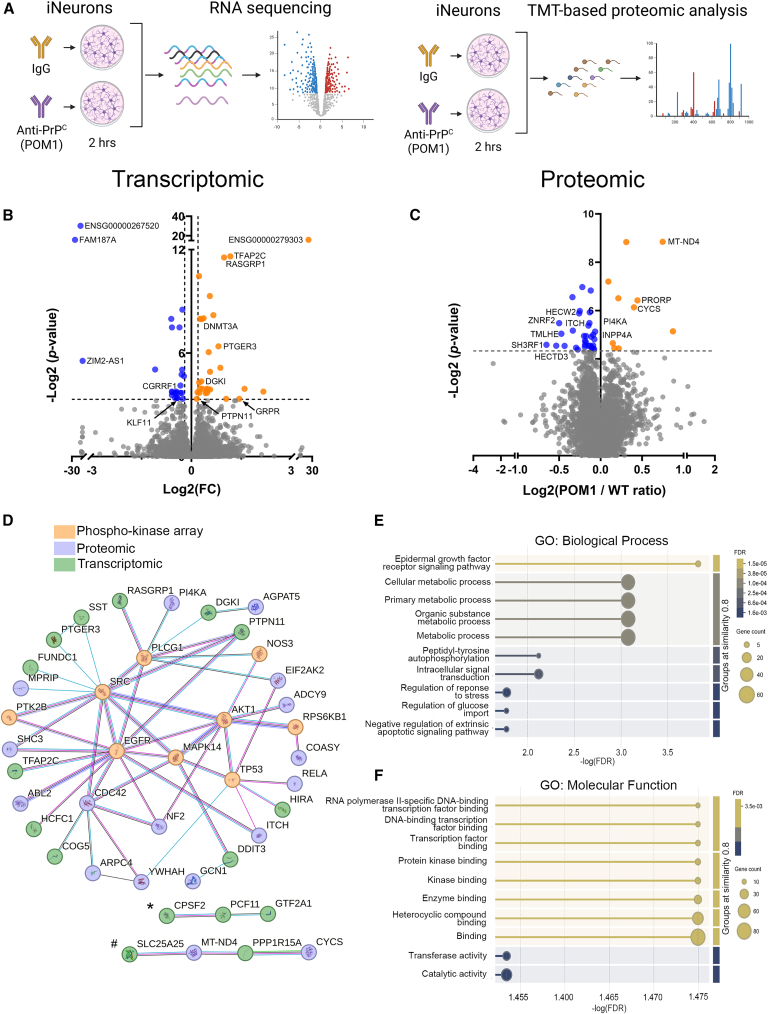
Multiomic profiling of iNs following POM1 exposure (A) Schematic representation of RNA sequencing and TMT-based proteomic analysis of iNs treated with POM1 or IgG for 2 h (*n* = 4 independent experiments). (B) Volcano plot shows genes altered following POM1 or IgG exposure. (C) Volcano plot shows peptides altered following POM1 or IgG exposure. (D) Predicted network of protein-protein interactions of significantly altered genes and proteins from transcriptomic (green), proteomic (blue), and phospho-kinase array analyses (orange). Three clusters are observed. EGFR is the central node (most predicted interactions) of the network 1. Symbols ^∗^ and # depict networks related to transcription factor and mitochondrial proteins, respectively. (E and F) Biological process and molecular function GO terms from protein-protein interaction analysis. For (B) and (C), blue = significantly decreased; orange = significantly increased.

To identify RNA alterations occurring in parallel with the increase in Arc, pERK1/2, and pPLC-γ1, we performed RNA-seq of iNs exposed to POM1 or IgG for 2 h (Figure 5A). The RNA-seq analysis revealed 53 differentially expressed genes (DEGs) (increased = 29, decreased = 24; *p* ≤ 0.1; ±25% change) (Figure 5B; Table S1). Of the 53 genes, 17% were involved in EGFR signaling and 15% were long non-coding RNAs (Figure 5B; Table S1). The downregulated EGFR-related genes include *CGRRF1*, an E3-ubiquitin ligase necessary for the proteasomal degradation of EGFR (Lee et al., 2019), as well as *KLF11*, a transcriptional repressor inhibited following EGFR-Ras-MEK1 signaling activation (Buttar et al., 2010; Ellenrieder et al., 2002). The upregulated EGFR-related transcripts included upstream regulators of EGF (*GRPR* and *PTGER3*) and EGFR transcription (*TFAP2C*), as well as mediators of EGFR signaling (*DNMT3A*, *PTPN11*, *RASGRP1*, and *DGKI*) (Figures 5A and 5B; Table S1). These DEGs implicate EGFR signaling as a cellular response to PrP^C^-ligand binding.

The RNA-seq revealed 12 DEGs (23%) also reported to be altered in the hippocampus of prion-infected mice (Ojeda-Juarez et al., 2022; Slota et al., 2022; Sorce et al., 2020) (Figure 5B; Table S1). These included *SST*, *LSM11*, *AATK*, *ABCD2*, *SATB2*, *GRPR*, *RNF138*, *HCFC1*, and *FUNDC1* (RML strain) (Slota et al., 2022; Sorce et al., 2020), and *SLC25A25* (22L strain) (Ojeda-Juarez et al., 2022). Transcripts of prostaglandin E3 receptor (*PTGER3*), which modulates EGFR ligands and worsens PrP^Sc^-mediated toxicity (Liu et al., 2024), were also increased.

### Triggering PrP^C^ induces proteomic alterations linked to metabolism

To probe how triggering PrP^C^ acutely impacts other proteins in an unbiased manner, we performed tandem mass tag (TMT) mass spectrometry on iNs following exposure to POM1 or IgG for 2 h (Figure 5A). We quantified 4,391 proteins and found 43 differentially expressed (*n* = 10 increased and 33 decreased with POM1 exposure) (*p* ≤ 0.05) (Figure 5C; Table S2). Notably, there was an increase in mitochondrial proteins (MT-ND4, CYCS, and PRORP) and a decrease in ubiquitin E3-ligases (HECW2, ZNRF2, ITCH, HECTD3, and POSH). There was also a decrease in components of the phosphoinositide pathway, as well as a decrease in phosphatidylinositol 4-kinase (PI4K) and inositol polyphosphate-4-phosphatase type I A (INPP4A) peptides (Figure 5C), which would be expected to reduce the synthesis of secondary messengers, such as phosphatidylinositol 4-phosphate (PI4P) and phosphatidylinositol 3,4-bisphosphate (PtdIns(3,4)P2) (Balla et al., 2008; Shin et al., 2005). Together, the proteomics data suggest that POM1-triggered PrP^C^ impacts mitochondria, protein degradation, and metabolic pathways in iNs.

### Network analysis reveals three protein-protein interaction clusters

To identify possible protein-protein interactions from the altered transcriptional, phospho-kinase, and proteomic signatures following PrP^C^ stimulation with POM1, we uploaded all hits into the StringDB database. Three clusters emerged: (1) an EGFR signaling network, with EGFR as the central node, (2) a mitochondrial protein network, and (3) the GTF2A1 transcription factor and mRNA processing network (Figure 5D). A gene ontology (GO) enrichment analysis of the biological process pathways revealed “EGFR signaling pathway” as the top altered pathway (false discovery rate = 0.00015) (Figure 5E), while the molecular function pathways revealed “protein kinase binding” as enriched (fourth most significant; false discovery rate = 0.0335) (Figure 5F), collectively suggestive of EGFR and signaling having a central role.

### Phospho-PLC-γ1 and phospho-EGFR are altered in late stages of prion disease

**Figure 6:**
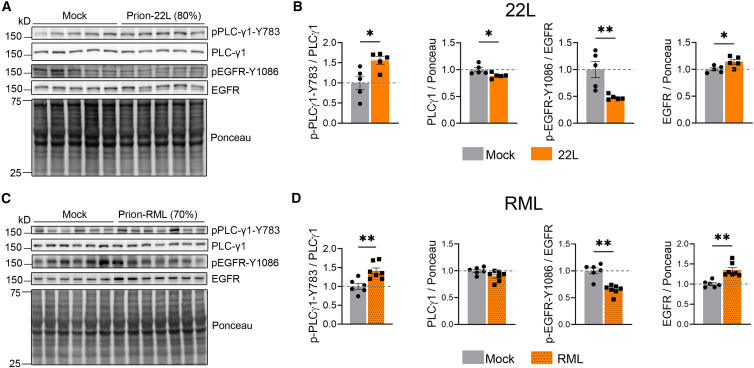
PLC-γ1 and EGFR phosphorylation are altered in prion-affected brain (A,C) Immunoblotting and (B,D) quantification of proteins from the cerebral cortex of prion-infected mice collected at the onset of clinical signs [timepoints, 22L: 80% (120 d.p.i); RML: 70% (123 d.p.i)], as compared to mock (uninfected brain)-inoculated controls (mock: *n* = 5–6; 22L: *n* = 5; RML: *n* = 7). Data are represented as fold change compared to mock samples. Shown is the mean ± SE. One-way ANOVA with Tukey’s MCT. ^∗^*p* ≤ 0.05; ^∗∗^*p* ≤ 0.01.

To determine whether PLC-γ1 and EGFR signaling are also altered in prion-infected mice, we inoculated two cohorts of WT mice with prions (22L and RML strains) or uninfected brain homogenate (mock), and analyzed cerebral cortex at the start of clinical signs (70%–80% of disease progression). Previous studies have shown minimal neuronal death at this stage (Hilton et al., 2013; Makarava et al., 2024; West Greenlee et al., 2016). We observed a marked increase in pPLC-γ1-Y783 (strain 22L: 56 ± 16% and RML: 40 ± 14%) compared with mock-inoculated controls (Figure 6). There was also a marked decrease in pEGFR-Y1086 (22L: 52 ± 5% and RML: 35 ± 9%) (Figure 6). These findings from prion-infected mice suggest that the signaling alterations observed in human iNs also occur *in vivo*, supporting the relevance of the acute POM1-iN model in revealing PrP^C^-linked signaling pathways.

Taken together, these data support a model in which triggering neuronal PrP^C^ with a pathogenic ligand leads to the internalization of PrP^C^ in a dynamin-dependent manner, activating PLC-γ1, which cleaves phosphatidylinositol 4,5-bisphosphate (PIP_2_) into inositol-1,4,5-trisphosphate (IP_3__)_. IP_3_ promotes Ca^2+^ release from ER stores, triggering downstream responses including Arc and the phosphorylation of ERK1/2.

## Discussion

GPI-anchored proteins localize to lipid rafts and interact with cell surface receptors to initiate intracellular signaling (Zhang and Fujita, 2024). Here we used proteomics, RNA-seq, and a phospho-kinase array to identify the cell signaling cascade initiated by a pathogenic PrP^C^ ligand, POM1, in human iNs. By integrating these unbiased techniques, we identify increased phosphorylation of PLC-γ1 and ERK1/2 at their activation sites, and an induction of Arc. Given that activated PLC-γ1 initiates Ca^2+^ release, which increases pERK1/2 through Ras-MAPK signaling and induces Arc (Peng et al., 2010), these data suggest that increased Ca^2+^ may be fundamental to acute PrP^C^-mediated cell-signaling.

POM1 stimulation of PrP^C^ induced PLC-γ1-Y783 phosphorylation in human iNs within 2 h. Additionally, pPLC-γ1-Y783 phosphorylation was increased in the cortex of prion-infected mice at the onset of clinical signs. PLC-γ1 is phosphorylated and activated by receptor tyrosine kinases, including EGFR (Kim et al., 1990; Meisenhelder et al., 1989; Vega et al., 1992). Activated PLC-γ1 catalyzes the cleavage of PIP_2_ into IP_3_ and diacylglycerol, and IP_3_ promotes Ca^2+^ release from the ER (Newton et al., 2016). Chronic activation of PLC-γ1, however, may result in a prolonged increase in cytosolic Ca^2+^ (Tao et al., 2023), which has been linked to neurotoxicity (Ferreiro et al., 2008; LaCasse et al., 2008; Ruiz et al., 2009). For example, prolonged ER Ca^2+^ release following NMDA, Aβ_1-40_, or prion peptide (106–126) exposure of mouse neurons exacerbates oxidative stress and mitochondrial membrane depolarization, promoting apoptosis and contributing to excitotoxicity (Ferreiro et al., 2006, 2008; Ruiz et al., 2009). The increased pPLC-γ1-Y783 may indicate that PrP^C^-mediated PLC-γ1 activation contributes to a chronic, pathological cycle of Ca^2+^ dysregulation in prion disease.

We found increased Arc in the frontal cortex of familial and sporadic prion disease-affected patients, indicative of neuronal hyperactivity. Congruent with our observation of increased Arc, patients in early- to mid-stages of sCJD show increased functional connectivity in the ventral and dorsal default mode network by fMRI, suggesting increased synchronized neuronal activity (Paoletti et al., 2022). Similarly, Arc is also increased in the frontal cortex of AD patients (Braak III-VI) (Wu et al., 2011), and AD patients with mild cognitive impairment also show elevated neuronal activity by fMRI (Celone et al., 2006; Dickerson et al., 2005; Targa Dias Anastacio et al., 2022). Furthermore, Aβ oligomers trigger an Arc response in rat hippocampal neurons (Lacor et al., 2004), which parallels our observations of increased Arc in PrP^Sc^-exposed mouse neurons. Thus, early neuronal hyperactivity may be a shared feature of prion disease and AD.

We found that ERK1/2 phosphorylation in POM1-treated iNs was dependent on dynamin-mediated endocytosis. This finding is consistent with work by Caetano and colleagues, who showed that dynamin-mediated internalization of PrP^C^ is required for stress-inducible protein 1 (STI1)-mediated ERK1/2 phosphorylation (Caetano et al., 2008). Interestingly, we found that Arc induction was also dependent on dynamin-mediated endocytosis, suggesting that PrP^C^ signaling from endosomes may drive neuronal activity. Determining whether PrP^Sc^ induces neuronal hyperactivity and signaling from the endosome will be important to investigate.

The GO analysis of transcriptomic and proteomic changes in POM1-treated iNs suggests a role for the tyrosine kinase, EGFR. In support of this role, we found that POM1 decreases the phosphorylation of EGFR at Y1086, a Grb2-binding site, suggestive of reduced EGFR activity after POM1 exposure. We observed a similar reduction in pEGFR-Y1086 in the prion-infected mouse brain, consistent with disrupted EGFR signaling. Groveman and colleagues showed a decrease in EGFR in *PRNP*^−/−^ human neural stem cells, together with increased cellular senescence, suggesting a link between EGFR and PrP^C^ (Groveman et al., 2023). Notably, EGFR-PrP^C^ interactions have been reported (Atkinson et al., 2019; Llorens et al., 2013; Martellucci et al., 2018). Factors that may regulate a PrP^C^-EGFR interaction include expression levels of both proteins, trafficking of EGFR to the cell surface and between membrane microdomains, and the availability of EGFR ligands. How EGFR activity is altered and may be linked to Ca^2+^ dysregulation in prion disease remains to be determined.

We used human iNs to investigate early signaling events following PrP^C^ stimulation, and found POM1-iNs as a robust, human-relevant model for the discovery of PrP^C^-induced signaling pathways. Notably, the increases in Arc and aberrant signaling observed in iNs were also detected in human and mouse prion-affected brain, respectively. We also recognize the limitations of this approach. Although we observe an increase in pPLC-γ1 and pERK1/2, together with an Arc induction and reduced pEGFR, the impact on neuronal health remains unclear. Moreover, our study models acute PrP^C^ stimulation, whereas prion disease develops over years. Determining the temporal dynamics of PLC-γ1 activation in neurons, the neuronal subpopulation affected, and the long-term consequences of chronic PLC-γ1 activation will require further investigation. Additionally, PLC-γ1 and EGFR are expressed in microglia and astrocytes (Liu et al., 2006; Qu et al., 2015), and it will be important to determine whether PrP^C^ stimulation triggers similar downstream signaling in glial cells. Finally, although Arc is a well-established marker of neuronal activity (Lyford et al., 1995; Waltereit et al., 2001), additional functional assays to measure synaptic plasticity and network excitability would be important to understanding the role of PrP^C^ signaling in neuronal hyperactivity and circuit-wide dysfunction in prion disease.

In summary, we show that PrP^C^ stimulation rapidly induces PLC-γ1 and ERK1/2 phosphorylation, as well as Arc expression, in human excitatory neurons. These results suggest that PrP^C^ functions as an upstream modulator of intracellular signaling with potential consequences for Ca^2+^ homeostasis. We propose that in prion disease, PLC-γ1 increases intracellular Ca^2+^, which triggers kinase-driven ERK1/2 activation and Arc translation, promoting neuronal survival and synaptic downscaling at weaker spines. Identifying the potential transmembrane co-receptors of PrP^C^ that drive PLC-γ1 activation, and the long-term consequences of chronic PLC-γ1-mediated signaling, will be important for determining the proximal causes of synaptic degeneration in prion disease.

## Resource availability

### Lead contact

Requests for resources, reagents, and additional information should be directed to the corresponding author, Christina J. Sigurdson (csigurdson@ucsd.edu).

### Materials availability

All unique reagents generated in this study are available from the corresponding author upon request.

### Data and code availability

• The data that support the findings of this study are either provided as supplemental information, deposited in publicly available repositories (RNA-seq data available from the GEO depository, accession number GSE327330; proteomic data available from the ProteomExchange depository, accession number PXD068396), or are available from the corresponding author upon request.
• This paper does not report original code.
• Any additional information required to reanalyze the data reported in this paper is available from the corresponding author upon request.

## Acknowledgments

We thank the National Prion Disease Pathology Surveillance Center (NPDPSC) for prion typing, and all patients and their families for participating in the research. Microscopy and image analysis were performed at the Nikon Imaging Center at UC San Diego. RNA-seq data were generated at the UC San Diego IGM Genomics Center. We thank Dr. Adriano Aguzzi for generously providing the *Prnp*^−/−^ (ZH3) mice. We thank Drs. Peng Guo and Richard Sánchez for support on microscopy experiments, Dr. Kristen Jepsen for support on RNA-seq experiments, Samantha Flores for technical assistance, the animal care staff at UC San Diego for excellent animal care, Jeff Metcalf, and the laboratory of Dr. Robert Rissman for providing patient samples. This study was supported by the 10.13039/100000002National Institutes of Health grants NS069566, NS076896, NS2033955, and NS105498 (C.J.S.), 5T32AG066596 (D.O.-J.), S10OD026929 (UC San Diego IGM Genomics Center), NS136112 (S.L.G.), AG062429 (UC San Diego Shiley-Marcos Alzheimer’s Disease Research Center), R01 AG031189 (M.D.G.), R56 AG055619 (M.D.G.), R01 AG062562 (M.D.G.), 5R01MH100175-11 (J.R.Y.), 1R01AG075862-03 (J.R.Y.), R01 AG077046-03 (J.R.Y.), and the UC President’s Postdoctoral Fellowship Program (D.O.-J.).

## Author contributions

C.J.S., D.O.-J., and S.L.G. conceptualized and designed experiments; D.O.-J., G.F., E.R., A.J.R., and K.S. performed experiments and analyzed the data; D.B.M. performed mass spectrometry experiments; C.J.S., D.O.-J., S.L.G., M.D.G., X.C., and J.R.Y. contributed to method design, data analysis, and interpretation; D.O.-J. and C.J.S. wrote the manuscript with input from all authors.

## Declaration of interests

Dr. Geschwind has consulted for Adept Field Consulting (Backbay Consulting), Ascel Health LLC, Anderson Boutwell Traylor, Best Doctors Inc., Biohaven Pharma Inc., Bioscience Pharma Partners, LLC (BPP), Clarion Consulting, First Thought Consulting, Grand Rounds Inc./UCSF Second Opinion Inc., Maupin Cox Legoy, Quest Diagnostics, Smith & Hennessey LLC, and Trinity Partners LLC. He has received speaking honoraria for various medical center lectures, Oakstone Publishing, and Wolters Kluwer. He has received past research support from Alliance Biosecure, CurePSP, the Tau Consortium, Quest Diagnostics, and NIH. Dr. Geschwind serves on the board of directors for San Francisco Bay Area Physicians for Social Responsibility and on the editorial board of Dementia & Neuropsychologia.

## STAR★Methods

### Key resources table

**Table:** 

REAGENT or RESOURCE	SOURCE	IDENTIFIER
**Antibodies**		

mouse anti-PrP POM2	Polymenidou et al. (2008)	N/A
mouse anti-PrP POM1	Polymenidou et al. (2008)	N/A
mouse anti-PrP 3F4	Barry and Prusiner (1986)	N/A
anti-GAPDH-HRP; Clone: GT239	GeneTex	Cat# GTX627408-01; RRID: AB_11174761
anti-phosphorylated EGFR-Y1068; Clone: D7A5D7	Cell Signaling Technology	Cat# 3777; RRID: AB_2096270
anti-EGFR; Clone: D1D4J	Cell Signaling Technology	Cat# 54359; RRID: AB_2799458
anti-phosphorylated ERK1/2-T202/Y204; Clone: D13.14.4E	Cell Signaling Technology	Cat# 4370; RRID: AB_2315112
anti-ERK1/2	Cell Signaling Technology	Cat# 4695; RRID: AB_390779
anti-phosphorylated PLC-γ1-Y783	Cell Signaling Technology	Cat# 14008; RRID: AB_2728690
anti-PLC-γ1	Cell Signaling Technology	Cat# 5690; RRID: AB_10691383
anti-Arc/Arg3.1	Proteintech	Cat# 16290-1-AP; RRID: AB_2151832
anti-phosphorylated p70 S6K-T389; Clone: 108D2	Cell Signaling Technology	Cat# 9234; RRID: AB_2269803
anti-p70 S6K	Cell Signaling Technology	Cat# 9202; RRID: AB_331676

**Biological samples**		

fCJD and GSS patient brain tissue	National Prion Disease Pathology Surveillance Center	https://case.edu/medicine/pathology/research/national-prion-disease-pathology-surveillance-center/cjd-research/biospecimen-request-cjd-research
sCJD patient brain tissue	UCSF Memory and Aging Center	https://memory.ucsf.edu/research-trials/professional/neurodegenerative-disease-brain-bank
Control brain tissue	Shiley-Marcos Alzheimer’s Disease Research Center	https://neurosciences.ucsd.edu/centers-programs/adrc/professionals/resources/index.html

**Chemicals, peptides, and recombinant proteins**		

Essential 8 Supplement	Thermo Fisher Scientific	Cat# A1517001
StemPro Accutase	Thermo Fisher Scientific	Cat# A1110501
GlutaMax^TM^	Thermo Fisher Scientific	Cat# 35050061
B-27^TM^ Plus Supplement	Thermo Fisher Scientific	Cat# A3582801
N2 Supplement	Thermo Fisher Scientific	Cat# 17502048
B-27^TM^ Supplement	Thermo Fisher Scientific	Cat# 17504044
BDNF	Thermo Fisher Scientific	Cat# 450-02
NT3	Thermo Fisher Scientific	Cat# 450-03
Doxycycline	Millipore Sigma	Cat# D9891
Y-27632	Cayman Chemical	Cat# 10005583
Mouse laminin	Thermo Fisher Scientific	Cat# 23017-015
Non-essential amino acids	Thermo Fisher Scientific	Cat# 11140050
cOmplete^TM^ Mini	Roche	Cat# 11836170001
PhosStop™	Roche	Cat# 04906837001
Benzonase™	Millipore Sigma	Cat# 70664
Dynasore™	Cayman Chemical	Cat# 14062
PI-PLC	Millipore Sigma	Cat# P5542

**Critical commercial assays**		

RNeasy Micro kit	Qiagen	Cat# 74004
TruSeq Stranded mRNA Sample Prep Kit	Illumina, Inc	Cat# 20020594
Proteome Profiler human phospho-kinase array	R&D Systems	Cat# ARY003C
LookOut Mycoplasma PCR Detection kit	Millipore Sigma	Cat# MP0035
TMT 10-plex isobaric label	Thermo Fisher Scientific	Cat# 90110

**Deposited data**		

Raw and analyzed TMT proteomics	This paper	ProteomExchange: PXD068396
Raw and analyzed RNA-seq	This paper	GEO: GSE327330

**Experimental models: Cell lines**		

WTC11 (human iPSC, Tet-ON 3G Ngn2)	Wang et al. (2017)	N/A

**Experimental models: Organisms/strains**		

Mouse: C57BL/6J	The Jackson Laboratory	JAX: 000664; RRID:IMSR_JAX:000664
Mouse: ZH3 *Prnp*^−/−^	Nuvolone et al. (2016)	N/A
Prion Strain: 22L	Li et al. (2022)	N/A
Prion Strain: RML	Chandler (1961)	N/A

**Software and algorithms**		

MultiGauge software	Fujifilm	http://lifescience.fujifilm.com/
Prism 10.5.0 (774)	GraphPad Software, Inc.	https://www.graphpad.com/
nf-core RNA-seq pipeline (3.13.2)		https://doi.org/10.5281/zenodo.1400710
Proteome Discoverer 2.5	Thermo Scientific	Cat# PROTEOMEDISC3

### Experimental model and study participant details

#### Human prion disease cases and controls

The 10 fCJD (all E200K) and 4 GSS (1 P102L, 3 F198S) cases were obtained from the National Prion Disease Pathology Surveillance Center at Case Western Reserve University (Table 1). Autopsy-verified, aged (control) brain samples were obtained from the Neuropathology Brain Bank of the Shiley-Marcos Alzheimer’s Disease Research Center at the University of California, San Diego (Table 1). The control brains were devoid of significant neurodegenerative disease pathology (postmortem) and disease-related cognitive impairment at the antemortem assessment proximate to death. All brain tissues used were de-identified samples collected at autopsy (NIH Office of Human Subjects Research Protections, exemption 4).

The sCJD brain tissues (fresh frozen) used in this study were obtained from a prospective study of sCJD patients evaluated between July 2015 and 2018 at the University of California, San Francisco (UCSF) Memory and Aging Center clinical research program for rapidly progressive dementia (RPD) (Table 1). All patients with sCJD had extensive clinical testing, including brain MRI, CSF analysis for 14-3-3, neuron-specific enolase (NSE), total tau, RT-QuIC (most cases) and were classified antemortem as probable sCJD by UCSF clinical and radiologic diagnostic criteria (Geschwind et al., 2007; Staffaroni et al., 2017). Cases were diagnosed postmortem as definite sCJD by immunoblotting and immunohistochemistry (Kretzschmar et al., 1996).

#### Approval of animal studies

All animal studies were approved by the Institutional Animal Care and Use Committee at UC San Diego. Protocols were performed in accordance with good animal practices, as described in the Guide for the Use and Care of Laboratory Animals published by the National Institutes of Health.

#### Prion inoculation of mice

C57BL/6J mice (6–8 weeks old) were anesthetized with ketamine and xylazine, and inoculated into the left parietal cortex with 30 μL of 1% prion-infected brain homogenate (22L or RML) prepared from terminally ill mice or 1% mock brain homogenate prepared from uninfected mice. The 22L and RML strains are mouse-adapted prions originally derived from sheep scrapie and characterized by diffuse prion aggregates, synaptic and neuronal loss, and glial activation in the brain (Chandler, 1961; Cunningham et al., 2005; Mallucci et al., 2003, 2007). Mice were maintained under pathogen-free conditions on a 12:12 light/dark cycle, and with access to standard laboratory chow and water *ad libitum*.

Prion-infected or mock-inoculated mice (n = 5–7) were euthanized at the beginning of clinical signs (22L: 80% of disease progression, 120 days post inoculation (d.p.i); and RML: 70% of disease progression, 123 d.p.i). The timepoints were approximated based on previous studies using each strain, however, the actual time to terminal disease may differ among cohorts. The cortex was dissected and flash-frozen in liquid nitrogen for immunoblotting.

#### Mouse primary cortical neuron culture

Primary cortical neurons were isolated from postnatal day (P)0–1 C57BL/6J or ZH3 *Prnp*^−/−^ mice pups. The cerebral cortices were dissected in dissection buffer (1 M MgCl_2_, 1 M CaCl_2_,1 M HEPES, and 2.77 M glucose), dissociated with 0.25% trypsin at 37°C for 20 min, treated with DNase, and triturated. Debris was removed by passing the cells through a 70-μm cell strainer. Cells were then centrifuged for 10 min and cultured in Neurobasal Plus media containing 2% B-27^TM^ Plus supplement and 1× GlutaMAX^TM^.

C57BL/6J or ZH3 *Prnp*^−/−^ cells at DIV 21 were treated with POM1 or IgG (33 nM). C57BL/6J cells at DIV 14 were treated with partially purified 22L or mock brain homogenate (100 nM) for the indicated time. Cells were lysed on ice in lysis buffer (10 mM Tris-HCl, 150 mM NaCl, 10 mM EDTA, 0.5% NP40, 0.5% DOC; pH 7.4 with Phos-STOP, nucleases [Benzonase], and protease inhibitors [cOmplete^TM^ Mini]. Triturated lysates were cleared by centrifugation at 10,000 × g for 10 min at 4°C. Proteins in the supernatant were quantified by BCA assay, and equivalent amounts of protein were electrophoresed through a 10% Bis-Tris gel and immunoblotted.

#### iPSC culture and neuronal differentiation

Human iPSCs from the wild-type genetic background (WTC11; male, Asian) line containing a Tet-ON 3G-controlled *Ngn2* transgene (Wang et al., 2017) were cultured in DMEM/F-12 (HAM) with Essential 8 Supplement and split at a 1:6 dilution when confluent using StemPro Accutase for dissociation. iPSCs from passages 12 to 19 were used for pre-differentiation.

iNs were differentiated with a two-step protocol (pre-differentiation and maturation) as previously described (Wang et al., 2017). Briefly, for pre-differentiation, iPSCs were plated into six-well plates coated with Matrigel, and incubated in knockout Dulbecco’s modified Eagle’s medium (KO-DMEM)/F12 medium containing N2 supplement (1X), non-essential amino acids (1× NEAA), mouse laminin (1 μg/mL), brain-derived neurotrophic factor (BDNF, 10 ng/mL), neurotrophin-3 (NT3, 10 ng/mL), and doxycycline (2 μg/mL) for 3 days. Day 1 media only was supplemented with Y-27632 (10 μM). The medium was changed daily.

For maturation (Day 4), pre-differentiated iN precursor cells were dissociated, counted, and plated at 650,000 cells/well in six-well plates coated with poly-L-lysine (PLL) in maturation medium containing 50% DMEM/F12, 50% Neurobasal-A medium, 0.5× B-27^TM^ supplement, 0.5× N2 supplement, 1× GlutaMax^TM^, 1× NEAA, doxycycline (2 μg/mL), mouse laminin (1 μg/mL), BDNF (10 ng/mL), and NT3 (10 ng/mL). Half the medium was changed weekly. Cells were handled in a sterile tissue culture hood, stored in a cell culture incubator with 5% CO2 at 37°C, and checked for bacterial contamination. Four passages of iPSC were tested for mycoplasma contamination with the LookOut Mycoplasma PCR Detection kit.

### Method details

#### Immunoblotting of mouse cerebral cortex

Cerebral cortices were homogenized in RIPA buffer (10% w/v) using a Beadbeater tissue homogenizer. Samples were lysed on ice for 30 min using 2% N-lauryl sarcosine in PBS with PhosSTOP, nucleases (Benzonase ), and protease inhibitors (cOmplete^TM^ Mini), then centrifuged at 2000 × g at 4°C for 10 min. Proteins in the supernatant were quantified by bicinchoninic acid (BCA) assay. Equal amounts of protein were reduced with DTT, electrophoresed through a 10% Bis-Tris gel (Invitrogen), and transferred to a nitrocellulose membrane by wet blotting. Membranes were incubated with primary antibodies overnight at 4°C, followed by incubation with an HRP-conjugated secondary antibody. The immunoblots were developed using a chemiluminescent substrate (ECL Supersignal West Dura or Femto) and visualized on a Fuji LAS 4000 imager. Densitometry analysis was performed using MultiGauge software.

#### Immunoblot analysis of fCJD, GSS, and sCJD brain samples

Immunoblotting of fresh frozen frontal cortex tissue from the sCJD, GSS, and E200K cases was performed as previously published with minor modifications (Ojeda-Juarez et al., 2022). Briefly, frontal cortex was homogenized in PBS using a Beadbeater tissue homogenizer. Protein levels were quantified using a BCA protein assay, 30 μg of protein per sample was lysed in PBS containing 2% sarcosyl, endonuclease (Benzonase), phosphatase inhibitors (PhosSTOP), and protease inhibitors (cOmplete^TM^ Mini) for 15 min at 37°C, and then centrifuged for 30 s at 18,000 × g to remove debris. DTT was added and samples were boiled for 5 min in LDS loading buffer (Invitrogen) and electrophoresed through a 10% Bis-Tris gel prior to transfer to a nitrocellulose membrane and immunoblotting.

#### PrP^Sc^ enrichment with sodium phosphotungstic acid

Concentration of PK-resistant PrP^Sc^ from E200K, GSS, and sCJD cortical patient samples by sodium phosphotungstic acid (NaPTA) precipitation was performed prior to western blotting as previously described (Wadsworth et al., 2001) with minor modifications. Briefly, 50 μL aliquots of 10% brain homogenate in an equal volume of 4% N-lauryl sarcosine in PBS were incubated for 30 min at 37°C with shaking, then digested with an endonuclease (Benzonase) followed by treatment with 100 μg/mL proteinase K (PK) at 37°C for 40 min. Then, samples were treated with 4% NaPTA/170 mM MgCl_2_ and protease inhibitors (cOmplete^TM^ Mini), incubated at 37°C for 30 min, and centrifuged at 14,000 rpm for 30 min at 25°C. The pellets were resuspended in 0.2% N-lauryl sarcosine prior to electrophoresis and immunoblotting.

#### Treatment of iNs with POM1 or IgG

After 6–8 weeks of maturation, the iNs were treated with the anti-PrP antibody POM1 (IgG), a single-chain variable fragment (scFv) of POM1, or a nonspecific IgG (all at 33 nM unless otherwise indicated). For inhibition of dynamin-mediated endocytosis, iNs were treated for 15 min with Dynasore^TM^ (100 μMl) prior to scFv POM1 stimulation. For cleavage of the GPI-anchor of PrP^C^, iNs were incubated with PI-PLC (0.5 u/mL) for 2 h at 37°C.

#### Partial purification of prion oligomers

Prion-infected and uninfected 10% brain homogenates in PBS were lysed by adding an equal volume of 2% N-lauryl sarcosine in PBS supplemented with Benzonase and 1 mM MgCl_2_ (final concentration). Lysates were incubated for 30 min at 37°C with mixing, and centrifuged at 18,000 g for 60 min at 4°C. The pellets were carefully rinsed with PBS, centrifuged at 18,000 g for 10 min at 4°C, re-suspended in PBS, and heated to 65°C for 30 min in preparation for cell culture. The PrP^Sc^ concentration was measured for each preparation by quantifying levels via immunoblotting against a standard curve from serially diluted recombinant PrP.

#### RNA-seq of iNs

For RNA-seq, 6 - 8 week-old iNs were treated with PBS, POM1 (33 nM), or IgG (33 nM) for 2 h. Samples were lysed in RLT buffer and RNA isolated using the RNeasy Micro kit following the manufacturer’s protocol. Total RNA was assessed for quality using an Agilent Tapestation 4200, and samples with an RNA Integrity Number (RIN) greater than 8.5 were used to generate RNA sequencing libraries using the TruSeq Stranded mRNA Sample Prep Kit. Samples were processed following the manufacturer’s instructions. Resulting libraries were multiplexed and sequenced with 100 base pair (bp) single reads (SR100) to a depth of approximately 25 million reads per sample on an Illumina NovaSeq X Plus. Samples were demultiplexed using bcl2fastq Conversion Software (Illumina, San Diego, CA). QC and RNA-seq were conducted at the IGM Genomics Center, University of California, San Diego, La Jolla, CA.

#### RNA-seq data processing

Fastqs files were processed by the Center for RNA Technologies and Therapeutics at UC San Diego using the nf-core RNA-seq pipeline (3.13.2) [https://doi.org/10.5281/zenodo.1400710]. Briefly, reads were adapter trimmed with cutadapt (3.4), mapped using STAR (2.7.9a), and quantified with Salmon (1.10.1) using annotations derived from Gencode V40. Differential expression analysis was performed with DESeq2 v1.32.0 on the raw counts matrix.

#### Phosphoprotein array studies of iNs

Phosphoproteins were detected using the Proteome Profiler human phospho-kinase array following the manufacturer’s instructions with minor modifications. Briefly, 6 - 8 week-old iNs treated with POM1 or IgG (33 nM) for 2 h were lysed using the kit lysis buffer (lysis buffer 6) with protease inhibitors (cOmplete^TM^ Mini). Membranes were incubated with 600 μg of protein overnight at 4°C, followed by incubation with detection antibody cocktails and streptavidin-HRP for detection. The immunoblots were developed using a chemiluminescent substrate (ECL Supersignal West Dura) and visualized on a Fuji LAS 4000 imager. Densitometry analysis was performed using MultiGauge software.

#### Mass spectrometry on iNs

6-week old iNs were treated with POM1 or IgG for 2 h, then lysed on ice using lysis buffer (10 mM Tris-HCl, 150 mM NaCl, 10 mM EDTA, 0.5% NP40, 0.5% DOC; pH 7.4) with PhosSTOP, nucleases (Benzonase), and protease inhibitors (cOmplete^TM^ Mini). Lysates were cleared by centrifugation at 10,000 × g for 10 min at 4°C. Proteins in the supernatant were quantified by BCA assay. 200 μg of samples were precipitated with methanol and chloroform, then digested with trypsin as previously described (Kawata et al., 2023). The digested peptides were desalted and then dried in a speed-vac. Each peptide sample was labeled with a unique TMT 10-plex isobaric label (Thermo Scientific) according to a published method (Zecha et al., 2019).

After the TMT-labeled samples were combined into one tube, 50 μg were removed for unmodified analysis. The TMT unmodified peptides were then each fractionated offline by high pH reverse-phase spin columns (Thermo Scientific) and analyzed on an Orbitrap Fusion Eclipse Tribrid mass spectrometer (Thermo Scientific). Samples were injected directly onto a 25 cm, 100 μm ID column packed with BEH 1.7 μm C18 resin (Waters). Samples were separated at a flow rate of 300 nL/min on an EasynLC 1200 (Thermo). Buffers A and B were 0.1% formic acid in water and 90% acetonitrile, respectively. A gradient of 1–25% B over 120 min, an increase to 40% B over 40 min, an increase to 100% B over 10 min, and held at 100% B for 10 min was used, for a 180 min total run time. Peptides were eluted directly from the tip of the column and nanosprayed directly into the mass spectrometer by application of 2.5 kV voltage at the back of the column. The Eclipse was operated in a data dependent mode. Full MS1 scans were collected in the Orbitrap at 120k resolution. The cycle time was set to 3 s, and within these 3 s, the most abundant ions per scan were selected for CID MS/MS in the ion trap. MS3 analysis with multinotch isolation (SPS3) was utilized for detection of TMT reporter ions at 60k resolution. Monoisotopic precursor selection was enabled, and dynamic exclusion was used with an exclusion duration of 10 s.

Proteome Discoverer 2.5 was used to search the MS/MS data to identify peptides, quantify the data, and perform statistical analysis. The MS spectra was searched using the Uniprot human protein database with isoforms (downloaded on 2023-2-16) and a common contaminant proteins list. The decoy database was the reverse of this Uniprot database to filter identifications to a 1% FDR. The peptides were allowed to have a maximum of two miscleavages. The static modifications searched were TMT on lysine and peptide N-terminal (+229.162932 Da) and cysteine carbamidomethylation (+57.021464 Da). Reporter ion distributions specific to the lot number of the TMT reagent were employed as correction factors.

#### Primary antibodies for immunoblotting

The following antibodies were used for immunoblotting: mouse anti-PrP [1:5,000, POM2 POM1 (Polymenidou et al., 2008); and 1:10,000, 3F4 (Barry and Prusiner, 1986)]; anti-GAPDH-HRP (1:5000); anti-phosphorylated EGFR-Y1068 (1:500); anti-EGFR (1:500 ); anti-phosphorylated ERK1/2-T202/Y204 (1:5000); anti-ERK1/2 (1:5000); anti-phosphorylated PLC-γ1-Y783 (1:5000); anti-PLC-γ1 (1:2000); anti-Arc/Arg3.1 (1:5000); anti-phosphorylated p70 S6K-T389 (1:2000); and anti-p70 S6K (1:5000).

### Quantification and statistical analysis

#### Statistical analysis

Analysis of immunoblotting data was performed using Prism software (GraphPad Software, Inc.). The number of mice, patients, or cells used for each experiment is provided in the figure legend. Data graphs show all samples analyzed and their SEM. No samples were excluded from this study. Comparisons between two groups were made by Mann-Whitney test or Welch’s *t* test for normally distributed data, whereas multiple groups were compared by Kruskal-Wallis test followed by Dunn’s MCT test. Spearman correlation was used to compute correlations between two groups. *p*-values ≤0.05 were considered statistically significant.

## Supplemental information

Data S1. Tables S1 and S2

Document S2. Article plus supplemental information
