# Supplementary material for: PrPC-facilitated cell signaling activates phospholipase Cɣ1 and triggers an Arc/Arg3.1 response in mouse and iPSC-derived human neurons
Source: Stem Cell Reports. 2026 May 14;21(6):102924. doi: 10.1016/j.stemcr.2026.102924 (PMC13261956; doi:10.1016/j.stemcr.2026.102924)
Supplement: Document S1. Figures S1–S6 [file mmc1.pdf]

Stem Cell Reports, Volume *21*

Supplemental Information

PrP^{C}-facilitated cell signaling activates phospholipase Cγ1 and triggers an Arc/Arg3.1 response in mouse and iPSC-derived human neurons

Daniel Ojeda-Juarez, Gail Funk, Daniel B. McClatchy, Emily Richards, Alexander J. Rajic, Katrin Soldau, Michael D. Geschwind, Xu Chen, John R. Yates III, Steven L. Gonias, and Christina J. Sigurdson

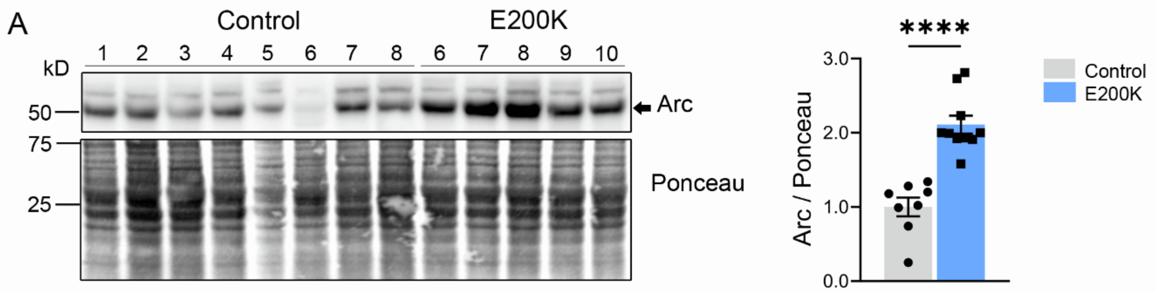

Figure S1. Arc is increased in human genetic prion disease, related to Figure 1. Immunoblotting of frontal cortex of **(A)** E200K familial prion disease (samples 6 – 10) (cases 1-5 are shown in Figure 1). Data is represented as fold change compared to control samples. Error bars = Standard Error of the Mean (SEM). Mann-Whitney test. ****P ≤ 0.0001.

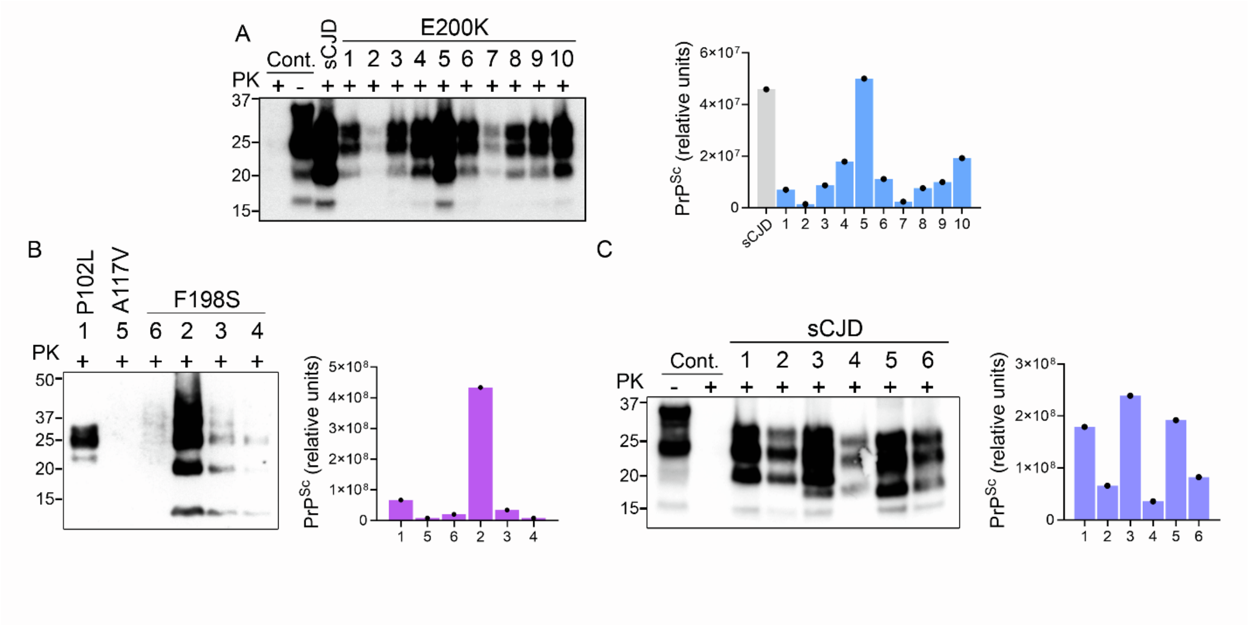

Figure S2. PrP^{Sc} levels in genetic and sporadic prion disease, related to Figure 1. Immunoblotting and quantification of PK-resistant PrP^{Sc} in the frontal cortex of **(A)** PrP-E200K (n = 10), **(B)** PrP-P102L and PrP-F198S (samples = 1-4; samples 5-6 represent additional cases not in included in the present study), and **(C)** sCJD patients (n = 6). Cont.: Non-prion disease control brain sample.

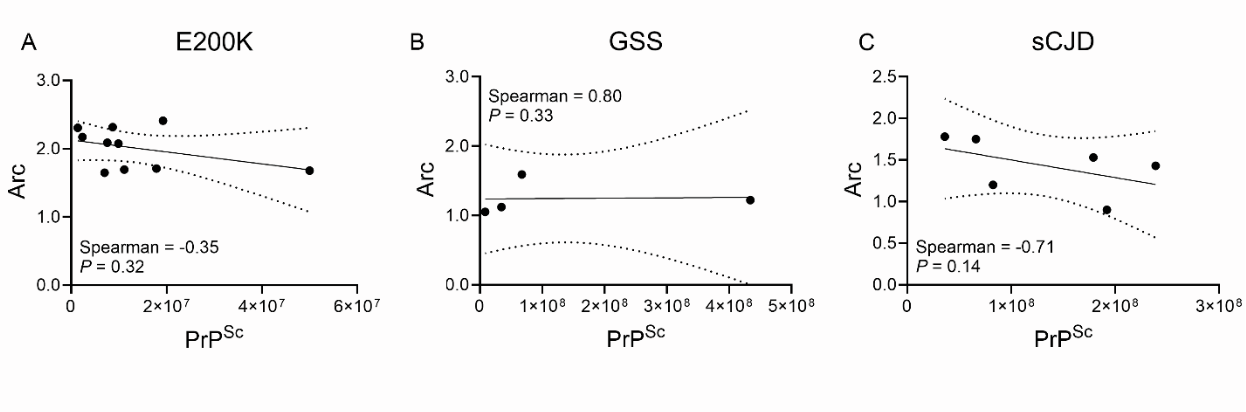

Figure S3. Correlation assessment between Arc and PrP^{Sc} levels in genetic and sporadic prion disease, related to Figure 1. Spearman correlation between Arc and PK-resistant PrP^{Sc} in the frontal cortex of **(A)** PrP**-**E200K, **(B)** PrP-P102L and PrP-F198S, and **(C)** sCJD patients.

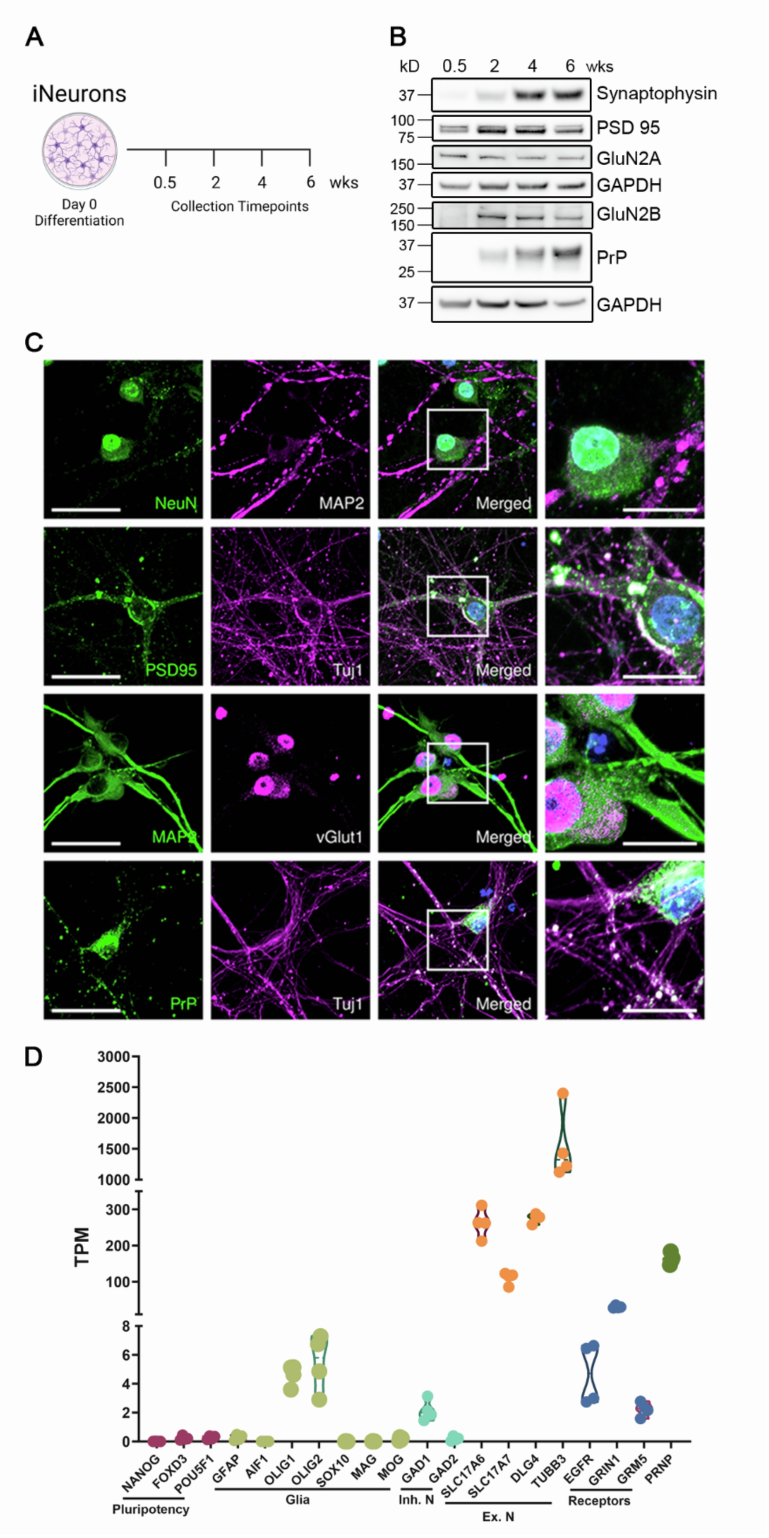
A
iNeurons
Day 0
Differentiation
0.5
2
4
6
wks
Collection Timepoints
B
kD
0.5
2
4
6
wks
Synaptophysin
PSD 95
GluN2A
GAPDH
GluN2B
PrP
GAPDH
37
100
75
150
37
250
150
37
25
37
C
NeuN
MAP2
Merged
PSD95
Tuj1
Merged
MAP2
vGlut1
Merged
PrP
Tuj1
Merged
D
TPM
3000
2500
2000
1500
1000
300
200
100
8
6
4
2
0
NANOG
FOXD3
POU5F1
GFAP
AIF1
OLIG1
OLIG2
SOX10
MAG
MOG
GAD1
GAD2
SLC17A6
SLC17A7
DLG4
TUBB3
EGFR
GRIN1
GRM5
PRNP
Pluripotency
Glia
Inh. N
Ex. N
Receptors

Figure S4. Characterization of neuronal and synaptic protein expression in human iNs, related to Figure 3. (A) Schematic showing the timepoints for iN collection. **(B)** Immunoblotting of synaptic proteins (presynaptic: synaptophysin, postsynaptic: PSD95), NMDA receptor subunits (GluN2A and Glu2NB), and PrP^C in iNs throughout maturation. **(C)** Representative immunofluorescent images of 6-week-old iNs showing expression of mature neuronal proteins (MAP2, Tuj1, and NeuN), vesicular glutamate transporters (vGlut), and a post-synaptic structural protein (PSD95) along with PrP^C localized to the soma and synapse. Scale bars = 50 μm (left three panels) and 20 μm (right panels). **(D)** RNA-seq analysis of 6-week-old iNs demonstrates the expression of excitatory (*SLC17A6*, *SLC17A7*, *DLG4*, *TUBB3*), glutamatergic (*GRIN1*, *GRM5*), and cellular prion protein (*PRNP*) transcripts (far right). N = 4 independent experiments.

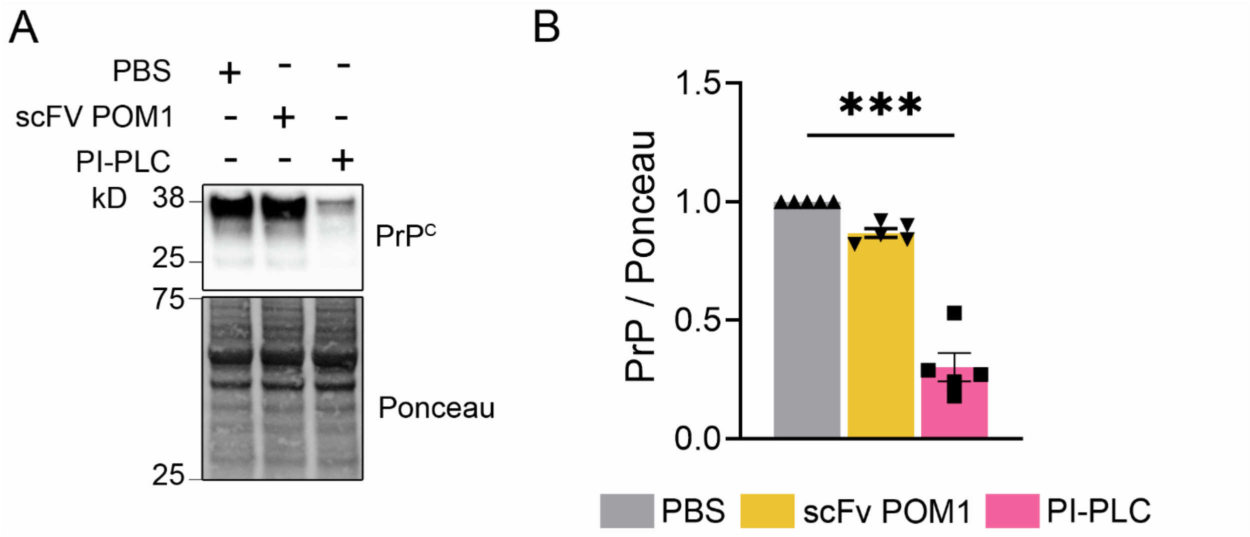

Figure S5. PrP^C is primarily located at the cell surface, related to Figure 3. (A) Immunoblot and **(B)** quantification of iNs treated with PI-PLC (0.5 U/mL) for 2 hours. N= 5 independent experiments. For Panel B, data is normalized to PBS for each experiment (PBS = 1). Error bars = SEM. Kruskal-Wallis test with Dunn's MCT. ***P ≤ 0.001.

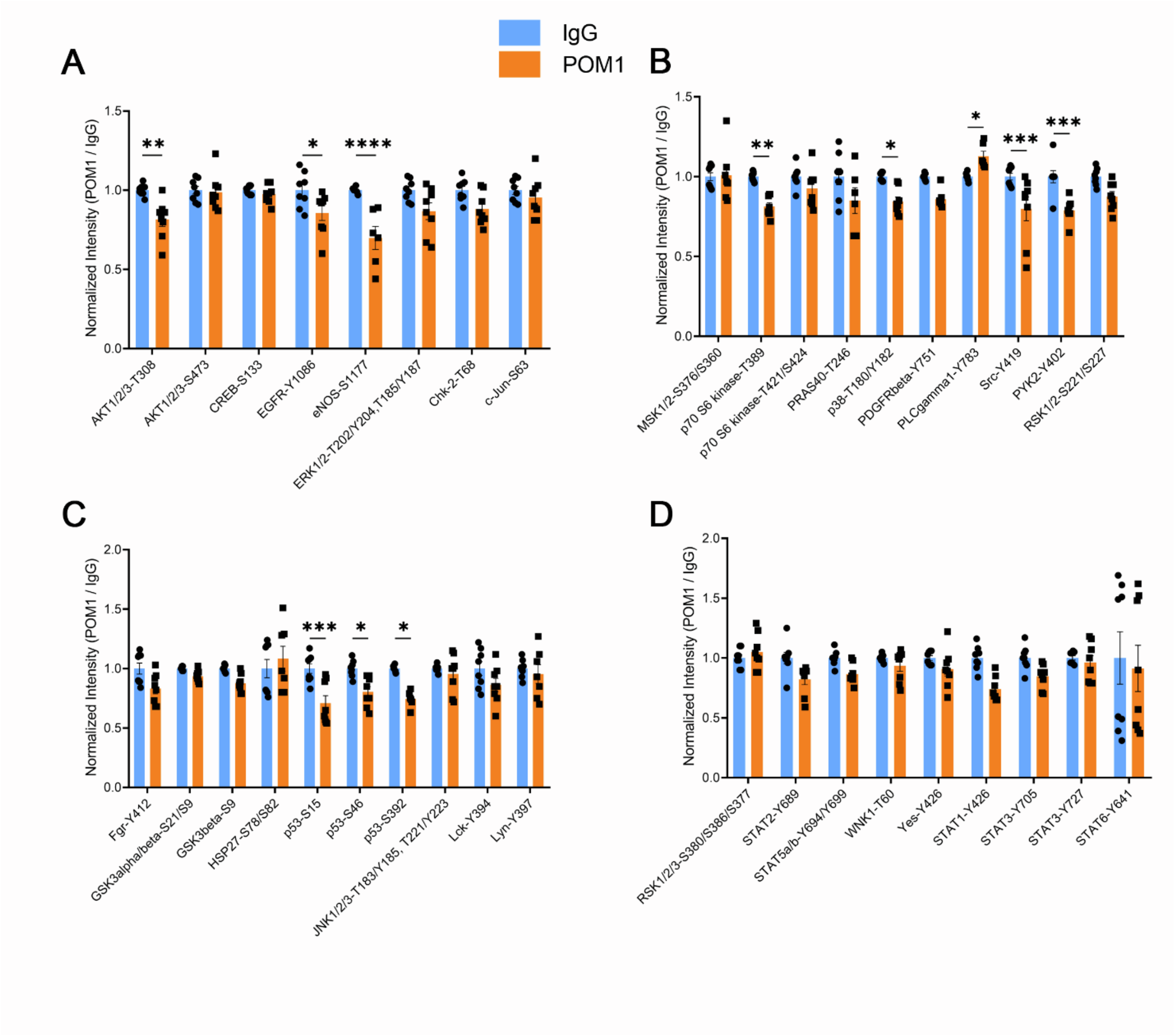

Figure S6. Phospho-kinase array of iNs treated with POM1 or IgG, related to Figure 4. (A - D) Quantification of all 37 phospho-sites in the phospho-kinase array following POM1 or IgG exposure for 2 hours. Data is normalized to the average of the two technical replicates of IgG for each experiment. Error bars = SEM. Two-way ANOVA with Sidak's MCT. *P ≤ 0.05; **P ≤ 0.01; ***P ≤ 0.001; ****P ≤ 0.0001.
